# Network spatial patterns and determinants of China’s hometown chambers of commerce

**DOI:** 10.1371/journal.pone.0331476

**Published:** 2025-09-05

**Authors:** Jiesong Gou, Jing Zhu

**Affiliations:** Institute of Western China Economic Research, Southwestern University of Finance and Economics, Chengdu, Sichuan, China; East China University of Science and Technology, CHINA

## Abstract

Based on the establishment data of provincial-provincial, city-city, provincial-city, city-provincial Hometown Chambers of Commerce (HCCs) in China by the end of 2022, this paper combines social network analysis and exponential random graph model to explore network spatial patterns and determinants of China’s HCCs. Findings indicate that: 1) Point degree centrality in eastern China is high, but spatial patterns vary across network types: provincial-provincial and provincial-city types exhibit a “rhombus-net” pattern, the city-city type follows a “small-ring-line” structure, and the city-provincial type forms a “large-ring-net” type. Guangdong, Fujian, Zhejiang, Jiangxi, and Shandong constitute core areas of China’s HCC network, and provincial-provincial and city-city types mainly flow within the core area and from core to periphery, while provincial-city and city-provincial types flow mutually between core and periphery areas. 2) Stronger government intervention and earlier HCC connections significantly inhibit both provincial-provincial and city-city HCC establishment. However, this inhibitory effect is not statistically significant for provincial-city and city-provincial types. Only city-city HCCs exhibit economic sensitivity, with less-developed origin cities favoring stronger operation cities, while other HCC types remain economically neutral. Road, dialect, and urban cluster distances significantly affect HCC establishment at the city-level place of operation but show no significant impact on the provincial-level place of operation. This research not only expands the theoretical perspective on the spatial study of social organizations but also provides scientific evidence for breaking down regional market fragmentation and optimizing cross-regional governance systems.

## 1. Introduction

As a sub-category of Chinese social organizations, Hometown Chambers of Commerce (HCCs) are voluntarily initiated and legally registered by enterprises from the same place of origin in the place of operation, and named after both the place of operation and origin [1]. Depending on the geographical scope of the place of operation and origin, HCCs are established through parallel and cross connections across regional, provincial, municipal and county levels, resulting in multiple types. For example, the Guangdong-Chengdu Chamber of Commerce is an association formed by entrepreneurs whose place of origin is Chengdu (municipal city) and whose place of operation is Guangdong (province).

Compared with other Chinese social organizations, HCCs exhibit unique spatial patterns. The networks formed by HCCs facilitate the flow of multiple elements such as personnel, capital, commodities and information [2]. HCCs play a significant dual role in guiding cross-regional economic cooperation and maintaining social order, demonstrating both economic and social values [3–4]. However, social organizations in China face historical sensitivity and management complexity [2]. Moreover, HCCs’ economic and social impact on the place of operation is relatively indirect, unlike the direct influence of local governments through investment attraction, public security management, and environmental pollution control—factors directly tied to officials’ performance evaluations [5]. Consequently, the relevant government departments lacking sufficient motivation, have an inadequate understanding of HCCs’ actual development. The network space of China’s HCCs exhibits significant hierarchical and geographical differentiation, and the underlying heterogeneous determinants remain systematically unexplored—a key focus of this study.

“Network space” refers to the topological structure formed by interconnections between geographical entities (e.g., provinces, cities) through flows of capital, information, or institutional ties [6–7]. Although the concept of “network space” has been widely applied in Chinese geographical research—examining innovation networks [8–9], tourism flows [10–11], energy networks [12–13], and high-speed railway mobility [14–15]—prior studies have overlooked its relevance to social organizations like HCCs. Unlike economic or infrastructure networks, HCC networks uniquely combine economic flows with cultural identity and institutional embeddedness, operating across heterogeneous administrative levels. This gap leaves their spatial dynamics unexplored. Existing research on China’s HCCs mainly focus on the determinants of HCC establishment [16–18], the social role of HCCs [19] and the economic role of HCCs [20–21]. However, most studies examine only provincial-level or localized HCCs, neglecting cross-level interactions such as provincial-provincial, city-city, provincial-city, and city-provincial HCCs. These HCC types differ in business coverage and driving factors due to their distinct administrative hierarchies. Furthermore, current research content predominantly adopts a temporal perspective (e.g., comparing regional economic benefits before and after HCC establishment [21]). By integrating geographical and flow space perspectives to analyze the cross-sectional network spatial patterns of China’s HCCs, this study will significantly enrich the field.

In view of this, based on the establishment data of provincial-provincial, city-city, provincial-city and city-provincial HCCs in China by the end of 2022, this paper firstly employs social network analysis (SNA) and geographic information systems (GIS) to explore the spatial patterns of the four types of HCC networks, including node network spatial patterns, core-periphery structures and network clustering. Node network analysis identifies highly connected nodes, revealing inflow/outflow hotspots within HCC networks; core-periphery analysis integrates these hotspots to delineate core versus peripheral areas; network clustering analysis further distinguish the clustering categories of different nodes in the network, identifying the directional flows of HCCs between core and peripheral areas. This helps determine whether core areas are formed through flow convergence or flow radiation. Next, this paper implements exponential random graph model (ERGM) to investigate the formation determinants of the four types of HCC networks. For each type of network, goodness-of-fit and convergence diagnostics are conducted to ensure the reliability of ERGM results.

Our study makes three contributions: (1) Previous studies have lacked a spatial perspective on analyzing HCCs. This paper pioneers comprehensively characterization of HCC networks’ topological structure, spatial differentiation, and cross-level interactions. (2) While prior studies have examined the impact on HCC establishment, most rely on qualitative case studies or single-type analyses. In contrast, our study conducts a systematic (covering multiple types of HCC) and heterogeneous (examining different HCCs separately) quantitative analysis of the determinants of China’s HCC establishment. (3) This research advances theoretical frameworks for spatial social organizations and empirically supports policies to reduce regional market fragmentation and optimizing cross-regional governance systems. It offers important policy insights for advancing the construction of a unified national market and the modernization of social governance in China.

The remainder of this paper is organized as follows: Section 2 develops the theoretical framework. Section 3 describes data sources and methodology, including SNA and ERGM specifications. Section 4 presents research results, analyzing network spatial patterns of HCCs and determinants of HCCs establishment. Section 5 discusses conclusions and policy implications based on key findings. Section 6 summarizes limitations and future directions.

## 2. Theoretical framework

“Network space” answers what the research object is, while “flow space” focuses more on answering why the network space pattern is like this. “Flow space” emphasizes the dynamic processes underpinning network formation, particularly the movement of political, economic and cultural elements that shape HCC establishment. It complements network space by revealing the mechanisms behind observed spatial patterns [6,22].

In terms of politics, local government intervention and institutional environment may affect HCC establishment. HCC formation may be influenced by government interventions in both the place of operation and origin, specifically reflected in government financial expenditures and administrative barriers [23]. While governments may increase financial expenditures to improve public goods for attracting foreign enterprises and facilitating HCC formation, stronger government intervention often leads to local protectionism—such as domestic firm subsidies and discriminatory taxes against foreign enterprises—which distorts market competition and hinders HCCs’ fair participation. Moreover, a sound institutional environment reduces policy distance between operation and origin places. Regional integration can weaken administrative barriers and ease cross-regional business operations [24], thereby enabling enterprises to establish HCCs.

In terms of economy, based on the orientation of economic factor flow and the spatial pattern of China’s economic development [25], local economic development levels may determine HCC connections. The cross-regional flow of enterprises depends on market conditions in both the place of operation and origin [1,3], and HCCs serve as key carriers of such flows. China’s social organization networks show a pronounced east-west imbalance, with eastern regions exhibiting significantly higher degree centrality than western areas [26]. Thus, when enterprises expand cross-regionally under market principles to access broader markets, they simultaneously drive demand for establishing HCCs.

In terms of culture, traditional cultural distance and historical factors may shape psychological motivations for establishing HCCs. Merchants and enterprises from other regions often differ from locals in customs and cultural roots, creating cultural distances and disadvantages in accessing local market information. HCCs leverage shared hometown identities (“hometown sentiment”) to facilitate information exchange [27]. Dialects, as key cultural markers [28], further drive this dynamic—merchants with distinct dialects from operation regions show stronger inclinations to form HCCs. Historically, HCCs trace back to Ming and Qing dynasty hometown associations [29]. Network preference attachment [30] makes new HCC connections dependent on existing ones. Early HCC development was spurred by state pilot policies, with successful cases creating institutional inertia that attracted more enterprises. Since China’s reform and opening-up, labor migration from inland to coastal areas enabled early migrant entrepreneurs to establish HCCs through trusted social networks. However, early HCCs concentrated in traditional industries faced industrial upgrading challenges [31]. Labor-intensive sectors hindered high-end factor agglomeration, while pollution-intensive industries trapped resources in low value-added activities, discouraging new HCC formation.

Given the geographical spatial characteristics of this network, establishing HCCs should consider road distance impacts on costs [32]. For certain HCCs, clear interactions exist between operation and origin places, where distance directly affects expenses for goods, personnel, and information flows. Thus, places with developed road infrastructure should be prioritized for HCC formation to optimize commercial and social outcomes. However, strong intervention from provincial or central governments—whether in origin or operation place—can override other determinants. This reflects the complex heterogeneity of driving factors across HCC types and administrative levels (both peer-level and cross-level).

## 3. Materials and methods

### 3.1. Materials

#### 3.1.1. Construction of HCC networks.

This study focuses on the spatial network structure of China’s HCCs. The spatial units of analysis include 333 prefecture-level administrative units (including autonomous prefectures, regions, and leagues) and 31 provincial-level units, excluding Hong Kong, Macao, and Taiwan. Throughout the paper, the term “city” refers to prefecture-level administrative region, and “province” refers to provincial-level administrative region. HCCs are classified into four types according to geographical connections between their place of operation and origin: provincial–provincial, city–city, provincial–city, and city–provincial HCCs.

The primary dataset included all officially registered HCCs established in mainland China up to the end of 2022, collected from the Tianyancha platform (https://www.tianyancha.com/). Data collection and analysis complied with Tianyancha’s Terms of Service (https://www.tianyancha.com/agreement). Specifically: Data were accessed only through publicly available pages or legally permitted interfaces (e.g., via manual search or approved API, if applicable); No automated scraping tools violating Tianyancha’s Robot Exclusion Protocol (robots.txt) were used; The dataset was anonymized/aggregated where necessary to protect sensitive commercial information.

Given the possibility of registration inconsistencies and classification errors, we implemented a multi-step data validation process to ensure reliability:

- **Duplicate removal:** Records with identical organization names, origin and destination locations, and registration dates were identified and removed.
- **Geographical standardization:** We harmonized location names across all entries using official administrative codes from the National Bureau of Statistics to eliminate errors caused by naming inconsistencies or regional aliases.
- **Error filtering:** Entries that had been revoked, canceled, or were clearly misclassified (e.g., origin and destination locations being the same) were removed—resulting in the exclusion of 107 records.
- **Cross-validation:** The remaining entries were manually checked and verified against multiple sources, including Baidu Baike and official websites of HCCs, to confirm registration legitimacy and organizational type.

After this cleaning and validation process, a total of 5,306 valid HCC records were retained for network construction.

Following the structural requirements of ERGM, we constructed four types of HCC networks. The provincial–provincial and city–city HCC networks were modeled as directed square matrices to reflect directional and potentially reciprocal flows. Self-ties were excluded from the matrices. To meet the ERGM requirement for square matrices, the original city–city network, which was 250 × 260 in size, was expanded to a 286 × 286 matrix by padding with non-relational nodes.

In contrast, the provincial–city and city–provincial HCC networks were modeled as bipartite (two-mode) undirected networks, representing unidirectional flows across different administrative levels. While these flows are inherently directional in nature, the modeling treats them as undirected due to the absence of triadic or clustering structural terms in the model. This simplification does not affect the validity of the ERGM results in this context.

All networks were converted into binary adjacency matrices (1 indicating the presence of a tie; 0 indicating absence). Matrix specifications are as follows: the provincial–provincial HCC network is a 31 × 31 square matrix with 675 connections; the city–city HCC network is a 286 × 286 square matrix with 2,071 connections; the provincial–city HCC network is a 236 × 31 rectangular matrix with 798 connections; and the city–provincial HCC network is a 30 × 270 rectangular matrix with 1,762 connections.

#### 3.1.2. Determinants of HCC networks.

The GDP, per capita GDP, and local general public budget expenditure data were all sourced from the official statistical databases of China from 2003 to 2022, including the following: China Statistical Yearbook (https://data.cnki.net/yearBook/single?nav=&id=N2024110295&pinyinCode=YINFN) and China Urban Statistical Yearbook (https://data.cnki.net/yearBook/single?id=N2025020156&pinyinCode=YZGCA).

The dialect data were derived from the book, Atlas of Languages in China (http://find.nlc.cn/search/showDocDetails?docId=8348547496026492250&dataSource=cjfd&query=%E4%B8%AD%E5%9B%BD%E8%AF%AD%E8%A8%80%E5%9C%B0%E5%9B%BE%E9%9B%86), including 9 dialects: Xiang, Gan, Hui, Wu, Zhongyuan Mandarin, Jianghuai Mandarin, Southwest Mandarin, Hakka, and others.

The urban cluster data were obtained from the 14th Five-Year Plan for National Economic and Social Development of the People’s Republic of China (https://www.gov.cn/xinwen/2021-03/13/content_5592681.htm), which mentions 19 urban agglomerations.

The road distance data were calculated based on the shortest intercity highway distances from the 2022 Amap (https://www.amap.com/) database. Our data collection and analysis complied with Amap’s Terms of Service (https://www.amap.com/terms). The data were obtained manually for academic research purposes only, without automated scraping or commercial use. The dataset was derived from Amap’s public route planning service and does not redistribute raw data from Amap.

### 3.2. Methods

#### 3.2.1. Point centrality.

The degree centrality module quantifies the influence level of nodes within the network. Nodes with higher degree centrality exhibit greater influence in the network space. In this study, the degree centrality of nodes is further divided into in-degree centrality and out-degree centrality.


$$C(n_{i})=\sum_{j}X_{ij}$$ (1)

Where: *C(n*_*i*_*)* is the in-degree/out-degree centrality of node *i*; *X*_*ij*_ is whether there is a connection between node *i* and node *j*, if there is a connection, its value is 1, otherwise it is 0. In order to facilitate the comparison between different types of networks, the in-degree/out-degree centrality is standardized, and the formula is as follows:


$$C_{1}^{*}(n_{i})=C(n_{i})/(d-1)$$ (2)

$$C_{2}*(n_{i})=C(n_{i})/(d-p)$$ (3)

where *C*_1_^*^(n_*i*_*)* and *C*_2_^*^(n_*i*_*)* are the normalized in-degree/out-degree centrality of node *i*. Equation (2) is applicable to provincial-provincial and city-city HCCs. *d* is the number of nodes in the network, and *d* − 1 is the maximum number of nodes that can be connected by HCCs in the network. Equation (3) applies to provincial-city and city-provincial HCCs. When the degree centrality of province is calculated, *p* is the number of network nodes of province; When calculating the degree centrality of city, *p* is the number of nodes in city network, and *d* − *p* is the maximum number of nodes that can be connected by HCCs in the network.

#### 3.2.2. Core-periphery structure model.

**Fig 1:**
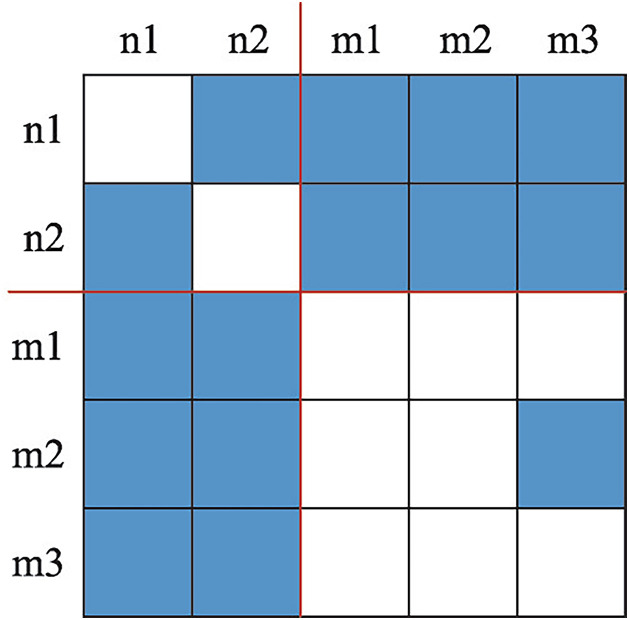
Matrix of core-periphery structure, (Note: Red lines partitioning the matrix into: core region (nodes n1 and n2) and periphery region (nodes m1, m2, and m3)).

The core-periphery structure model identifies nodes’ positional status (core vs. periphery) within networks. It employs graph-theoretic and relational-structure methods to group nodes into distinct areas and analyze inter-area relationships [33]. Abstracted into a matrix representation (Fig 1), this framework is characterized by: blue grid cells indicating HCC flows between node pairs, predominantly within core areas and between core-periphery. The matrix is partitioned by horizontal and vertical red lines into core are (containing nodes n1 and n2) and periphery area (comprising nodes m1, m2, and m3).

#### 3.2.3. Cohesive subgroups.

**Fig 2:**
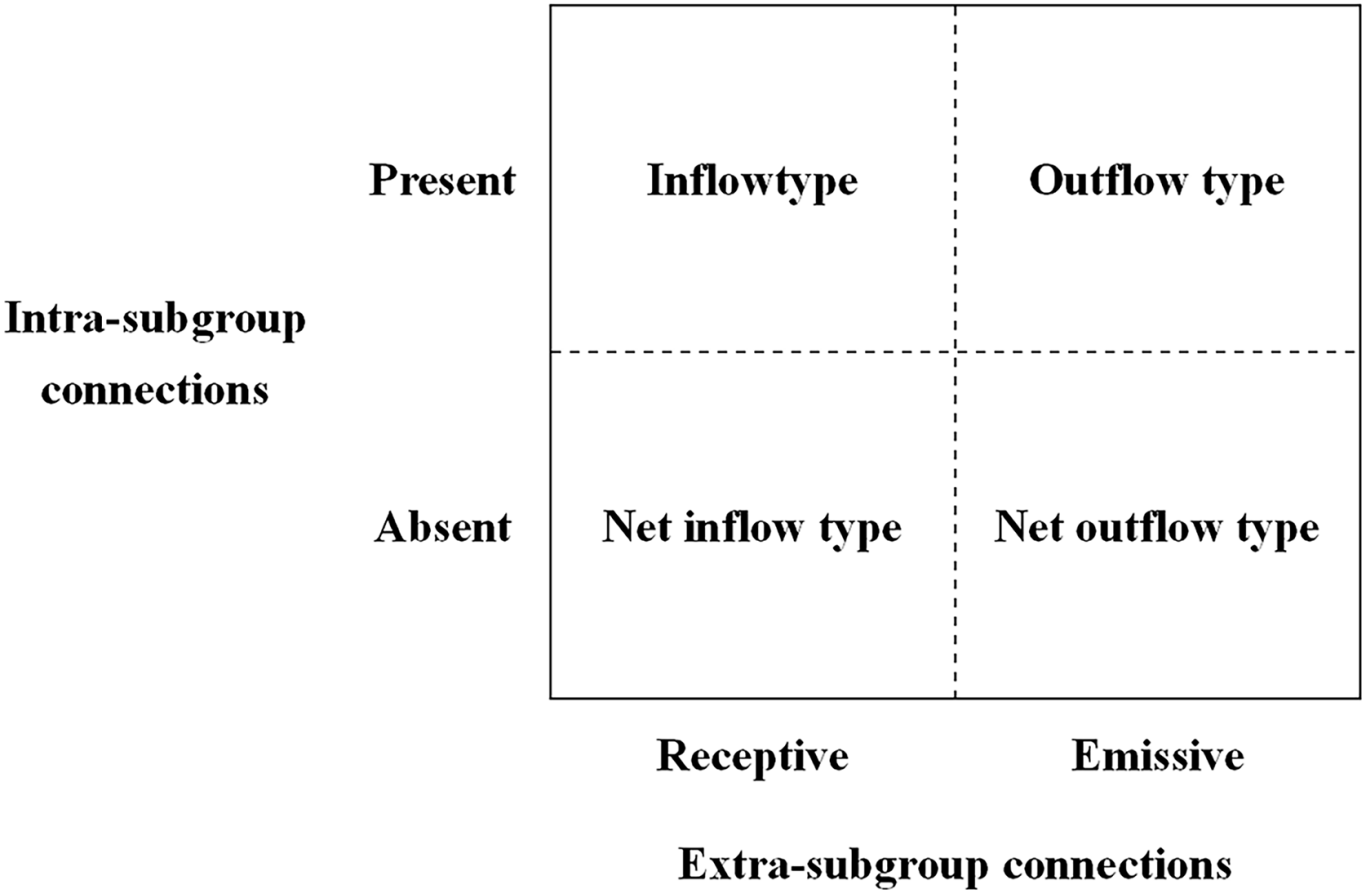
Types of cohesive subgroups.

The cohesive subgroup model classifies node clusters in networks and identifies HCC flow directions between core and periphery areas. Through correlation coefficient analysis, it evaluates pairwise node similarities and partitions nodes into subgroups. Subgroup relational flows are determined by intra- and inter-group density comparisons [34]. Comparative analysis of connection densities reveals four flow typologies (Fig 2): (1) Net inflow type: subgroups with negligible internal but dominant external inbound connections; (2) Net outflow type: subgroups with minimal internal but predominant outbound connections; (3) Outflow type: subgroups with established internal and dominant outbound connections; and (4) Inflow type: subgroups with strong internal and dominant inbound connections.

#### 3.2.4. ERGM.

(1) **Model instruction**

This study selects ERGM to analyze the determinants of network connection of China’s HCCs. We prioritize the ERGM framework because our dependent variable constitutes relational data (whether a HCC connection exists between two locations), and network modeling based on relational characteristics explicitly accounts for network dependencies (e.g., transitivity, reciprocity), accommodates complex structures (node attributes, node assortativity, network covariates), and yields interpretable coefficients (log-odds of tie formation) that traditional linear regression or QAP tests cannot comprehensively address.

ERGM is a statistical model that explains the observed characteristics of the macroscopic network by using hypothetical micro-network configuration [35]. The model assumes a pattern network with N nodes *G(N)*={*V*, *J*}, where *V*={1,2,..., *n*} denotes the node set; *J* ={(*i*, *j*):*i*, *j* ∈ *V*, *i* ≠ *j*} represents all possible node pairs. So for a given observation network *G* = {*V*, *E*}, which *E* indicates the existing edges in the observation network, we define a binary random variable *Y* to represent the elements in the *J*, if (*i*, *j*) ∈ *E*, *Y*_*ij*_ = 1, *Y*_*ij*_ = 0 otherwise. Thus, *P*(*Y = y*|θ) can be used to represent the probability that *y* occurs in the feasible set *Y* under the condition *θ*.


$$P(Y=\;y|\theta )=\frac{1}{k(\theta )}\text{exp}(\sum \beta g(x))$$ (4)

Where: *P*(*Y = y*|θ) represents the probability that there is connection between HCCs, which is 1 if there is connection between HCCs, otherwise it is 0. *g(x)* represents the influencing factors of network connections, including three types: endogenous structure of network, node and edge covariates.

(2) **Specification of network statistics**

The ERGM statistics include three categories: endogenous network structures, node covariates, and edge covariates (Table 1). Endogenous network structures are intrinsic variables primarily consisting of terms such as *Edges*, *Mutual*, *Ctriple*, *Degree (k)*, and *K-star (k)*. The selection of these endogenous structures is based on both characteristics of network objects and model fit. For instance, *Ctriple* is suitable for directed square matrices, while *Degree (k)* applies to undirected matrices like bipartite networks. Notably, when both *K-star (k)* and *Ctriple* are included in a model simultaneously, issues of collinearity may arise, making model convergence difficult in this paper.

**Table 1:** Statistic configurations in the ERGM.

Categories	Configurations	Instructions	Literature
**Endogenous structure**	*Edges*	Similar to the constant term of the econometric model, reflecting the network density.	[35–36]
	*Mutual*	The two places tend to move from one another.	
	*Ctriple* ^*1*^	The tendency for three places to form closed triangles in the network.	
	*Degree (k)* ^*2*^	Identifying the centrality structure of networks or capturing the heterogeneity in degree distribution.	
**Node covariates**	*Government intervention*	The proportion of public budget expenditure to the GDP in the place of operation or origin (The mean value for the period 2003–2022).	[23–24]
	*History of HCC* *establishment*	The time of establishing the first HCC in the place of operation or origin. If there is no HCC in the place of operation, it is marked as 0, 2022 is marked as 1, 2021 is marked as 2, and so on.	[29–31]
	*lnpergdp*	Log value of per capita GDP of the place of operation or origin (The mean value for the period 2003–2022).	[25–26]
**Edge covariate**	*lndistance*	The log value of the road distance between two places in 2022.	[32]
	*Dialect*	If two places are in the same dialect area, it is marked as 1; if not, it is marked as 0.	[27–28]
	*Urban cluster*	If two places are in the same urban cluster in 2022, it is marked as 1; if not, it is marked as 0.	[24]

Note: First, ^1^ indicates that the *Ctriple* is applicable solely to directed square matrices (specifically, provincial-provincial and city-city networks), while ^2^ denotes the *Degree (k)* restricted to bipartite matrices (namely, provincial-city and city-provincial networks). Second, since most provincial-level HCCs are connected from the provincial capital city, when provincial HCCs involve *Dialec*t and *Urban cluster* distance variables, attribute values of provincial capital city are used as proxies. Third, the **History of* HCC establishment* variable incorporates HCCs that had closed down or were unobserved by December 2022.

The exogenous variables comprise node and edge covariates, which in this study were selected based on established theoretical literature, including *Government intervention*, *History of HCC establishment*, *lnpergdp*, *lndistance*, *Dialect*, and *Urban cluster*. Among these, *Dialect* and *Urban cluster* are categorical variables, while others are continuous variables. All continuous variables mentioned above were winsorized at the 1% level to mitigate the influence of extreme values. And all exogenous variables exhibit Variance Inflation Factors (VIF) below 3, indicating the absence of severe multicollinearity.

(3) **Model testing**

Based on the constructed statistics in this study, we employed a stepwise addition approach to examine the ERGM fitting results to prevent model degeneration. This study determined variables retention through four criteria: First, whether the model converged after adding variables; second, whether the model’s goodness-of-fit improved (i.e., decreased AIC and BIC values) post-addition; third, whether the added variables’ parameters passed significance testing (p-value < 0.10), a threshold chosen to balance Type I/II error risks given smaller sample sizes, account for network sparsity, and facilitate cross-type comparative analysis; finally, upon completing the model fitting, we provided goodness-of-fit plots and convergence diagnostic diagrams for each types of HCC networks to ensure model reliability.

## 4. Research results

### 4.1. Network spatial patterns of China’s HCC

#### 4.1.1. Overall network characteristics.

The calculated network structure indicators of China’s HCCs are presented in Table 2, revealing their overall network structure characteristics. First, provincial-provincial networks exhibit high density (0.726), small diameter (2), and average path length (1), indicating single-step connections between any two provinces. High clustering (0.759) and reciprocity (0.525) demonstrate strong regional cohesion and bidirectional interactions, conforming to “small-world” network properties [37]. Second, city-city networks show low density (0.025) and large diameter (10), requiring approximately four steps for inter-city connections. The clustering coefficient (0.227) exceeds network density (0.025), suggesting connections concentrate around central hub cities with limited peripheral interactions. The near-zero reciprocity (0.002) confirms predominantly unidirectional flows, exhibiting “core-periphery” structures [33].

**Table 2:** The overall network descriptive statistics of China’s HCCs in 2022.

Indicators	Province-province	City-city	Province-city	City-province
**Network density**	0.726	0.025	0.011	0.020
**Network diameter**	2	10	1	1
**Average path distance**	1.274	3.787	1	1
**Clustering coefficient**	0.749	0.227	0	0
**Reciprocity coefficient**	0.525	0.002	0	0
**Nodes**	31	286	267	300
**Connections**	675	2071	798	1762

Note: This table presents descriptive statistics for four HCC networks. Due to cross-level administrative properties of provincial-city and city-provincial networks, the network diameter, average path distance, clustering coefficient and reciprocity coefficient are all fixed values.

#### 4.1.2. Spatial patterns of node networks.

**Fig 3:**
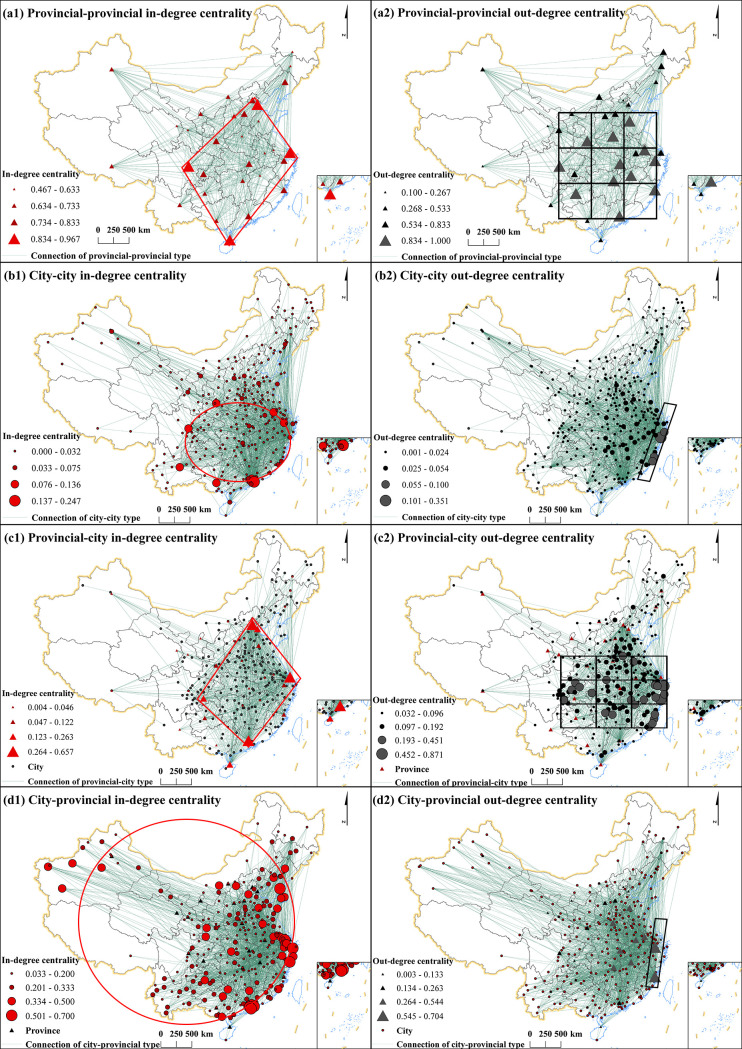
Map of degree centrality of China’s HCC networks in 2022. (Note: In this graph, provincial nodes are represented by triangles and city nodes by circles; in-degree is shown in red and out-degree in black. For the sake of visual clarity, the direction arrows of the various HCC connections are omitted. The direction can be determined by the in-degree and out-degree of nodes; The base map was obtained from the Standard Map Service of the Ministry of Natural Resources of China (http://bzdt.ch.mnr.gov.cn/), with approval number GS(2019)1823, and the base map boundaries remain unmodified. The same applies to Fig 4–5.).

In this section, UCINET 6 is used to calculate degree centrality metrics (in-degree and out-degree) for HCC networks, followed by standardization. Then, ArcGIS visualized nodal centrality distribution (Fig 3). Using the Jenks natural breaks classification, centrality values were segmented into four tiers: star degree, high degree, medium degree and low degree.

Fig 3 (a1, a2) show degree centrality of provincial-provincial networks. In terms of in-degree centrality, Tianjin (0.967), Sichuan (0.933), Shanghai (0.9) and Hainan (0.867) are star degree nodes. In terms of out-degree centrality, there are many star degree nodes, and Fujian, Henan, Hubei, Hunan, Jiangsu, Jiangxi, and Shandong all reach maximum degree centrality (1.000), indicating complete reciprocal HCC flows, followed by Guizhou, Shanxi and Zhejiang (0.967) and Guangdong and Sichuan (0.900). The spatial coupling of high in/out-degree centrality nodes forms a “rhombus-net” pattern.

The degree centrality distributions of city-city networks are shown in Fig 3 (b1, b2). For in-degree centrality, Shenzhen (0.247) is the star degree node, and the high nodes, ranked from high to low, are Chengdu (0.136), Kunming (0.132), Xi’an (0.124), Nanning (0.109), Dongguan (0.103), Nanjing (0.102) and Hangzhou (0.089). For out-degree centrality, Wenzhou (0.351) is the star degree node, followed by Taizhou (0.100), Quanzhou (0.089), Putian (0.754) and Fuzhou (0.054) from high to low. The spatial coupling of centrality nodes exhibits a “small ring-line” pattern.

The degree centrality distributions of provincial-city networks are shown in Fig 3 (c1, c2). In terms of in-degree centrality, Beijing (0.657), Shanghai (0.648) and Guangdong(0.466) are the star degree node, and node from high to low in turn for Tianjin (0.263), Hainan (0.237) and Chongqing (0.212). In terms of out-degree centrality, Wenzhou (0.871) and Ningbo (0.677) are the star degree nodes, and there are 21 cities in the high nodes, which are widely distributed in eight provinces such as Zhejiang, Fujian, Sichuan, Hunan, Hubei, Jiangxi, Shandong and Jiangsu. This spatial configuration exhibits a “rhombus-net” pattern.

The degree centrality distributions of city-province networks are shown in Fig 3(d1, d2). For in-degree centrality, Shenzhen (0.700), Ningbo (0.633), Zhuhai (0.600), Dalian (0.600), Wenzhou (0.566), Zhongshan (0.533) and Suzhou (0.533) are star high nodes. Out-degree centrality peaks in Fujian (0.704) and Zhejiang (0.622), followed by Henan (0.544), Shandong (0.507), Jiangsu (0.500), Jiangxi (0.478), Hunan (0.433), Anhui (0.407) and Hubei (0.404). This spatial configuration exhibits a “large ring-network” pattern.

#### 4.1.3. Spatial patterns of core-periphery network.

**Fig 4:**
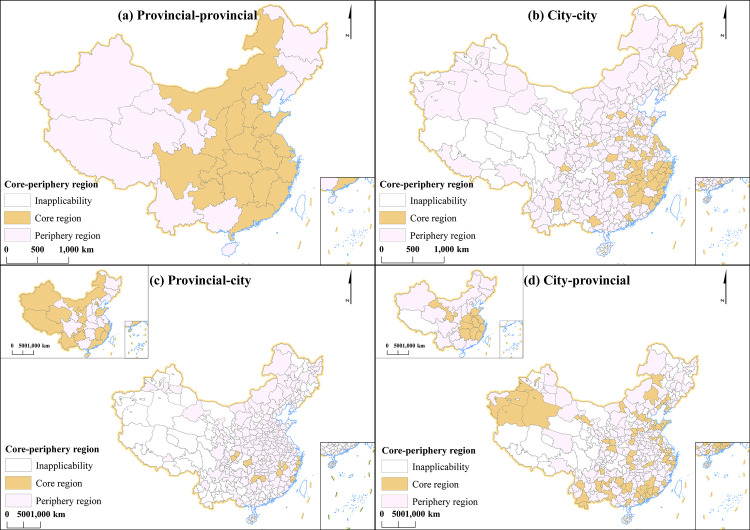
Map of core-periphery of China’s HCC networks in 2022. (Note: Figs (a) and (b) are maps of core-peripheral areas within the same administrative unit. Figs (c) and (d) are maps of core-peripheral areas across administrative units.).

This section uses software UCINET 6 Core and Periphery discrete module to identify the network node position in China’s HCC networks, and then spatial patterns of core-periphery networks are illustrated through ArcGis (Fig 4).

The 19 provincial-provincial core areas dominate the network (61.3% of 31 total; Fig 4(a)); City-city networks exhibit 53 core areas, all located east of the “Hu Huanyong Line”, with dense clusters in southeastern China and scattered provincial capitals (Fig 4 (b)). These patterns reflect an “eastern club” spatial structure, characterized by frequent HCC interactions among eastern regions; Provincial-city networks contain 30 core areas: provincial-level cores concentrate in coastal/border provinces, while city-level cores scatter across southern China (Fig 4 (c)); City-provincial networks show 29 cores, with provincial-level cores clustered in eastern/central regions and city-level cores dispersed nationwide (Fig 4 (d)). Compared to provincial/city types, cross-level cores demonstrate stronger western penetration—likely driven by policies like the pairing assistance program that incentivize eastern investments in western development.

In short, China’s HCC core-periphery structures exhibit significant spatial heterogeneity, blending proximity (eastern clusters) and isolation (western outliers). Guangdong, Fujian, Zhejiang, Jiangxi, and Shandong have always occupied core areas, which constitute the primary core hubs of China’s HCC networks.

#### 4.1.4. Spatial patterns of network clustering.

**Fig 5:**
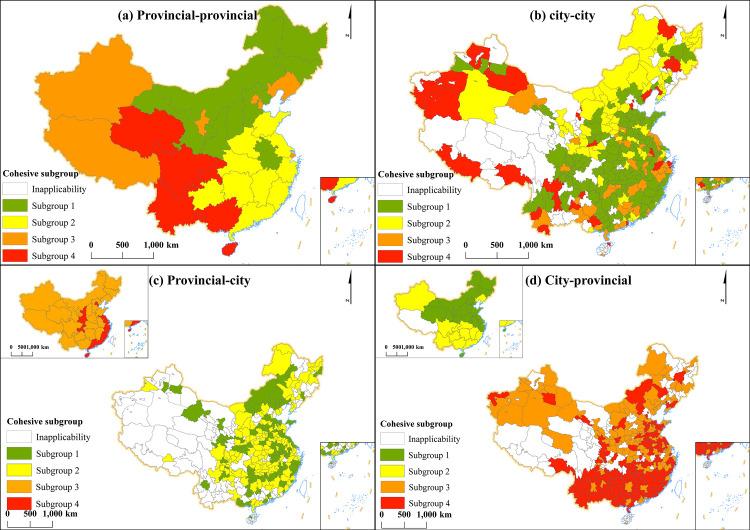
Map of cohesive subgroups of China’s HCC networks in 2022. (Note: Fig 5(a,b) are maps of cohesive subgroup within the same administrative unit. Fig 5(c,d) are maps of cohesive subgroup across administrative units. Subgroups with the same name may have different types in different cohesive subgroup maps.).

In this section, the Concor module is used to divide network clustering subgroups of China’s HCCs through UCINET 6, and then network spatial patterns of the clustering subgroups are shown through ArcGis (Fig 5). According to the density value of network subgroups, the type of each subgroup can be determined, and the flow direction of different kinds of HCC in the core area and the periphery interval can be clarified (Table 3).

**Table 3:** Density matrix among subgroups of China’s HCC networks in 2022.

Province-province	Subgroup 1	Subgroup 2	Subgroup 3	Subgroup 4	City-city	Subgroup 1	Subgroup 2	Subgroup 3	Subgroup 4
Subgroup 1	0.696/0	0.788/1	0.857/1	0.875/1	Subgroup 1	0.028/1	0.054/1	0.021/0	0.079/1
Subgroup 2	0.962/1	0.978/1	1/1	1/1	Subgroup 2	0.006/0	0.017/0	0.005/0	0.029/1
Subgroup 3	0.518/0	0.286/0	0.548/0	0.381/0	Subgroup 3	0.026/1	0.059/1	0.042/1	0.096/1
Subgroup 4	0.313/0	0.600/0	0.595/0	0.800/1	Subgroup 4	0.008/0	0.045/1	0.017/0	0.054/1
**Province-city**	Subgroup 1	Subgroup 2	Subgroup 3	Subgroup 4	**City-province**	Subgroup 1	Subgroup 2	Subgroup 3	Subgroup 4
Subgroup 1	0	0	0.033/1	0.245/1	Subgroup 1	0	0	0.280/1	0.148/1
Subgroup 2	0	0	0.024/1	0.303/1	Subgroup 2	0	0	0.198/1	0.261/1
Subgroup 3	0	0	0	0	Subgroup 3	0	0	0	0
Subgroup 4	0	0	0	0	Subgroup 4	0	0	0	0

Note: This table shows density matrices of four types of China’s HCC cohesive subgroups. The header row represents subgroups receiving connections, and the header column represents subgroups sending connections. The values in front the symbol (/) are density values between subgroups sending connections and subgroups receiving connections, and in order to make the structure more clear, density values between subgroups are compared with corresponding global network density (Table 2). If the former is greater than the latter, the corresponding location density is 1; otherwise, it is 0.

Provincial-provincial subgroups 1 and 2 (Fig 5 (a)) exhibit high overlap with their core areas (Fig 4 (a)). Table 3 shows that provincial-provincial subgroup 1 represents a net outflow type, characterized by weak internal HCC connection density but strong inflows to the other three subgroups. Subgroup 2 demonstrates an outflow pattern with robust HCC inflows both internally and externally. Subgroups 3 and 4 correspond to net-inflow and inflow types respectively, primarily receiving external subgroup inflows. City-city subgroups 1 and 3 (Fig 5 (b)) similarly overlap significantly with their core areas (Fig 4 (b)). Table 2 identifies subgroups 1 and 3 as outflow types, while subgroup 2 is net-inflow and subgroup 4 inflow type in marginal regions. Minimal overlap occurs between provincial-city/city-province subgroups (Fig 5(c,d)) and core-periphery areas (Fig 4(c,d)). However, high relational density between subgroup types 1–2 and 3–4 indicates strong provincial-city connectivity.

In general, provincial-provincial and city-city HCCs mainly flow within core areas and core areas flows to periphery areas, while provincial-city and city-provincial HCCs flow mutually between core and periphery areas.

### 4.2. Determinants analysis

#### 4.2.1. ERGM empirical result.

The analysis results of determinants for the four types of HCCs are presented in Tables 4–7. Model 1 includes only endogenous structural variables, while Models 2 and 3 sequentially add node covariates and edge covariates to Model 1, respectively. During the variable addition process, we monitored changes in the *Edges* coefficient and Standard Error - including increased absolute coefficient values, reduced coefficient significance, sign reversal of coefficients, and inflated Standard Errors (SEs) – as indicators of potential model degeneracy. Variables causing degeneracy were subsequently eliminated to obtain the optimal Model 4. For instance, in Table 4, Model 2 introduces node covariates to Model 1, resulting in a non-significant *Edges* coefficient (1.929) with an inflated Standard Error (4.486), signaling severe model degeneracy. Through separate regressions of node covariates, we identified *lnpergdp* as poorly fitted and thus excluded it. Model 3 incorporates edge covariates but produces a significant yet extreme *Edges* coefficient (27.408, SE = 3.29), again indicating model degeneracy. Similar procedures were followed for Tables 5–7.

**Table 4:** ERGM empirical results of provincial-provincial HCCs.

Category	Variables	Model 1	Model 2	Model 3	Optimal Model 4
Endogenous structure	*Edges*	2.839*** (0.502)	1.929 (4.486)	27.408*** (3.290)	4.802*** **(0.634)**
	*Mutual*	−0.017 (0.240)	0.087 (0.248)	−0.327 (0.255)	**0.065** **(0.247)**
	*Ctriple*	−0.120*** (0.031)	−0.076** (0.029)	−0.151*** (0.023)	−0.086*** **(0.030)**
Node covariates of operation place	*Government intervention*		−1.476* (0.866)		−1.202* **(0.729)**
	*History of HCC establishment*		0.010 (0.021)		**0.005** **(0.020)**
	*lnpergdp*		−0.176 (0.240)		
Node covariates of origin place	*Government intervention*		−2.856*** (0.936)		−3.560*** **(0.794)**
	*History of HCC establishment*		−0.070*** (0.015)		−0.069*** **(0.014)**
	*lnpergdp*		0.411 (0.265)		
Edge covariates	*lndistance*			−1.673*** (0.219)	
	*Dialect*			0.219 (0.463)	
	*Urban cluster*			−2.092*** (0.577)	
AIC		1087	1061	1003	**1060**
BIC		1101	1104	1032	**1194**
N		675	675	675	**675**

Note: Dependent variable is provincial-provincial HCC connection, with 1 indicating a connection and 0 otherwise. BIC is larger than AIC, and the smaller the two values, the better the model fit. SEs appear in parentheses. Significance levels: * **p* *< 0.1, ** **p* *< 0.05, ***p < 0.01.

**Table 5:** ERGM empirical results of city-city HCCs.

Category	Variables	Model 1	Model 2	Model 3	Optimal Model 4
Endogenous structure	*Edges*	−3.865*** (0.029)	−3.513*** (0.745)	−2.563*** (0.435)	−2.569*** **(0.901)**
	*Mutual*	1.268*** (0.119)	1.270*** (0.119)	1.139*** (0.120)	1.150*** **(0.119)**
	*Ctriple*	0.627*** (0.065)	0.616*** (0.062)	0.581*** (0.066)	0.568*** **(0.061)**
Node covariates of operation place	*Government intervention*		0.286 (0.256)		**0.230** **(0.252)**
	*History of HCC establishment*		−0.032*** (0.004)		−0.035*** **(0.004)**
	*lnpergdp*		0.404*** (0.053)		0.401*** **(0.055)**
Node covariates of origin place	*Government intervention*		−0.707*** (0.257)		−0.759*** **(0.259)**
	*History of HCC establishment*		−0.039*** (0.005)		−0.042*** **(0.005)**
	*lnpergdp*		−0.349*** (0.056)		−0.354*** **(0.056)**
Edge covariates	*lndistance*			−0.102*** (0.031)	−0.065** **(0.030)**
	*Dialect*			0.375*** (0.064)	0.438*** **(0.068)**
	*Urban cluster*			0.503*** (0.051)	0.530*** **(0.050)**
AIC		19136	18958	18935	**18740**
BIC		19164	19042	18990	**18852**
N		2071	2071	2071	**2071**

Note: Dependent variable is city-city HCC connection, with 1 indicating a connection and 0 otherwise. BIC is larger than AIC, and the smaller the two values, the better the model fit. SEs appear in parentheses. Significance levels: * **p* *< 0.1, ** **p* *< 0.05, ***p < 0.01.

**Table 6:** ERGM empirical results of provincial-city HCCs.

Category	Variables	Model 1	Model 2	Model 3	Optimal Model 4
Endogenous structure	*Edges*	−1.888*** (0.044)	−0.950 (1.593)	−0.217 (0.493)	−4.717*** **(0.532)**
	*Degree (1)*	1.768*** (0.197)	1.550*** (0.199)	1.779*** (0.205)	1.546*** **(0.202)**
Node covariates of operation place	*Government intervention*		0.336 (0.496)		1.425*** **(0.381)**
	*History of HCC establishment*		0.171*** (0.010)		0.143*** **(0.009)**
	*lnpergdp*		−0.643*** (0.129)		
Node covariates of origin place	*Government intervention*		0.519 (0.346)		**0.227** **(0.277)**
	*History of HCC establishment*		0.062*** (0.008)		0.063*** **(0.008)**
	*lnpergdp*		0.110 (0.078)		
Edge covariates	*lndistance*			−0.120*** (0.035)	−0.109*** **(0.034)**
	*Dialect*			0.190 (0.169)	
	*Urban cluster*			0.084 (0.142)	
AIC		4975	4586	4960	**4603**
BIC		4989	4642	4994	**4651**
N		798	798	798	**798**

Note: Dependent variable is provincial-city HCC connection, with 1 indicating a connection and 0 otherwise. BIC is larger than AIC, and the smaller the two values, the better the model fit. SEs appear in parentheses. Significance levels: * **p* *< 0.1, ** **p* *< 0.05, ***p < 0.01.

**Table 7:** ERGM empirical results of city-provincial HCCs.

Category	Variables	Model 1	Model 2	Model 3	Optimal Model 4
Endogenous structure	*Edges*	−1.206*** (0.230)	−14.390*** (1.314)	2.866*** (0.525)	−5.538*** **(1.074)**
	*Degree (3)*	1.282*** (0.209)	1.299*** (0.215)	1.257*** (0.213)	1.253*** **(0.217)**
Node covariates of operation place	*Government intervention*		0.088 (0.255)		
	*History of HCC establishment*		−0.020*** (0.006)		−0.020*** **(0.006)**
	*lnpergdp*		0.347 (0.061)		**0.018** **(0.052)**
Node covariates of origin place	*Government intervention*		3.248*** (0.536)		
	*History of HCC establishment*		0.073*** (0.005)		0.055*** **(0.005)**
	*lnpergdp*		1.010*** (0.090)		0.628*** **(0.072)**
Edge covariates	*lndistance*			−0.287*** (0.037)	−0.246*** **(0.034)**
	*Dialect*			−0.348*** (0.114)	−0.190* **(0.110)**
	*Urban cluster*			−0.706*** (0.175)	−0.779*** **(0.181)**
AIC		8458	8133	8381	**8106**
BIC		8472	8189	8416	**8167**
N		1762	1762	1762	**1762**

Note: Dependent variable is city-provincial HCC connection, with 1 indicating a connection and 0 otherwise. BIC is larger than AIC, and the smaller the two values, the better the model fit. SEs appear in parentheses. Significance levels: * **p* *< 0.1, ** **p* *< 0.05, ***p < 0.01.

In provincial-provincial HCC ERGM analysis (Table 4), Model 4 shows: (1) The coefficients of *Government intervention* of operation and origin place are both negative and significant, indicating that stronger government intervention in both the place of operation and origin inhibits provincial-provincial HCC establishment. (2) The coefficient of *History of HCC establishment* of origin place is positive and significant, suggesting that earlier HCC establishment in the origin province reduces subsequent outflows of such HCCs from that province. This may be attributed to the low-end path dependence of early-stage industries, which face difficulties in transformation and upgrading, thereby weakening the local capacity to foster emerging business models and ultimately reducing the outflow potential of HCCs.

In city-city HCC ERGM analysis (Table 5), Model 4 shows: (1) The analysis reveals that *Government intervention* of origin place is significantly negative, indicating that weaker government intervention in origin cities drives local enterprises to establish HCCs elsewhere. (2) All coefficients of *History of HCC establishment* are significantly negative, suggesting that early-established HCCs attract fewer subsequent HCC formation. (3) The significantly positive *lnpergdp* of origin place and significantly negative *lnpergdp* of operation place demonstrate that less-developed cities tend to establish HCCs in more developed cities. (4) In edge covariates, the significantly negative *lndistance* confirms proximity preference in HCC linkages, while the positive *Dialect* coefficient reflects higher HCCs likelihood between linguistically similar cities—a pattern largely attributable to intra-provincial HCC connections, as evidenced by the significantly negative *Dialect* coefficient when excluding intra-provincial HCCs. The positive *Urban cluster* coefficient further underscores the facilitating role of shared urban agglomeration membership in HCC formation. The city-city HCC establishment faces not only challenges such as local protectionism and difficulties in industrial upgrading for enterprises but is also influenced by other factors that may affect micro-level market mechanisms. For instance, economic development levels impact commodity prices and labor wages, road distance affects transaction costs, and dialect distance influences the level of transactional trust. These micro-level market mechanisms directly shape the activities of enterprises and HCCs.

For provincial-city HCC ERGM networks (Table 6), Model 4 retains well-fitted variables: (1) The significantly positive *Government intervention* of operation place implies provinces with stronger government intervention more readily attract city-based enterprises to establish provincial-city HCCs. This reflects the siphoning effect of provincial-level administrative resources on city-level origin enterprises, where government interventions such as fiscal incentives, institutional facilitation, and political endorsement gradually attract external enterprises to establish HCCs. (2) Positive coefficients of *History of HCC establishment* for both inflow (province) and outflow (city) places demonstrate path dependence—provincial resources exert stable attraction, incentivizing cities to initiate sustained HCC flows once initial linkages form. The province-city HCC establishment follows a path-dependent historical trajectory, where early events trigger increasing returns to scale, thereby shaping long-term developmental pathways.

For city-provincial HCC networks (Table 7), Model 4 reveals: (1) the coefficient for *History of HCC establishment* of origin place is positive and significant, whereas *History of HCC establishment* of operation place exhibits a significant negative relationship. HCC outflows from provinces of origin demonstrate positive historical path dependence, while HCC inflows to destination cities is constrained by pre-existing HCC establishment, seemingly facing a bottleneck in industrial transformation. (2) The significantly positive *lnpergdp* of origin place and non-significant *lnpergdp* of operation place indicate city-provincial HCCs predominantly originate from economically advanced provinces. This aligns with Fig 3(d), where policy interventions (e.g., the Western Development Program and Aid-Xinjiang initiatives) drive eastern provinces to establish HCCs in western/border regions. (3) The three distance edge covariates all exert significant negative effects on HCC formation, demonstrating clear patterns of spatial, cultural, and institutional distance decay.

#### 4.2.2. ERGM testing.

**Fig 6:**
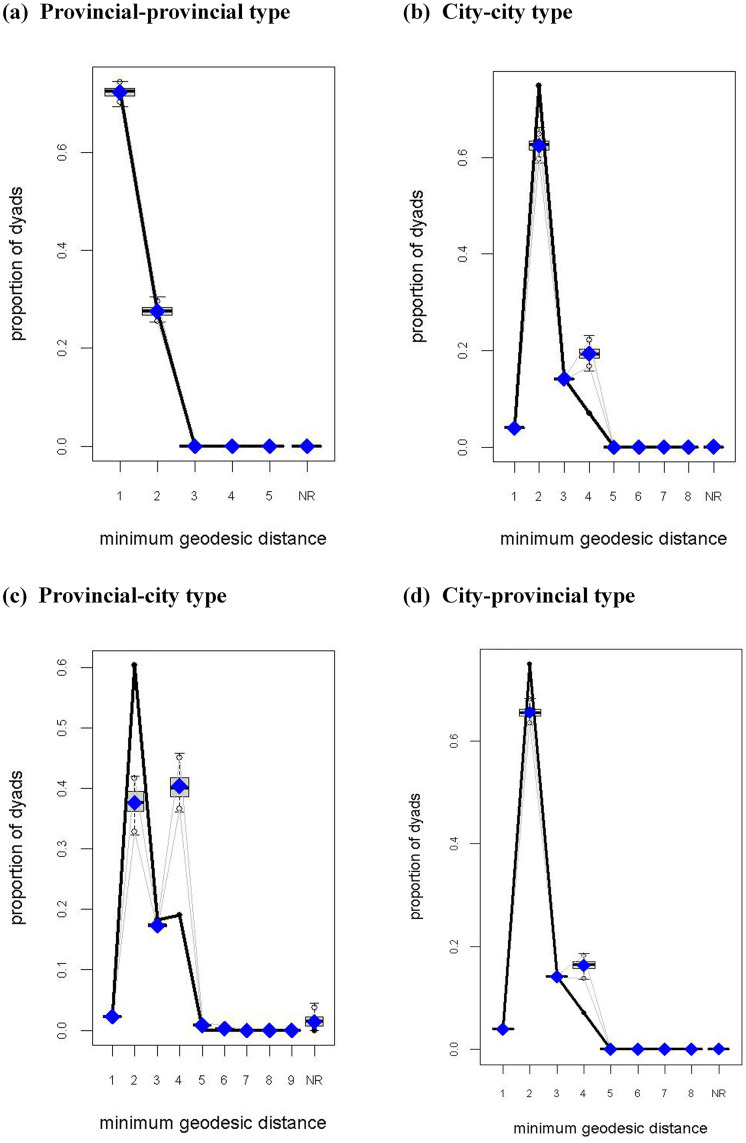
Goodness-of-fit diagnostics for the ERGM.

In the goodness-of-fit diagnostic plot (Fig 6), the black line (observed values) falls within the box-plot range (simulated values distribution), indicating that the shortest path distribution of the model-generated random networks aligns with the real network. Specifically: provincial-provincial model demonstrates the best fit, with observed and simulated values nearly overlapping completely (Fig 6(a)). City-city model show moderate fit quality, with minor deviations between observed and simulated values around a shortest path length of 2 and 4 (Fig 6(b)). The GOF test treats both provincial-city and city-provincial models as undirected bipartite networks, which theoretically allows for multiple minimum geodesic distance. In reality, the minimum geodesic distance between these two network types is exactly 1 (as shown in Table 2). Consequently, GOF statistics with minimum geodesic distance >1 should not be used as criteria for evaluating overall model fit (Fig 6(c, 6d).

**Fig 7:**
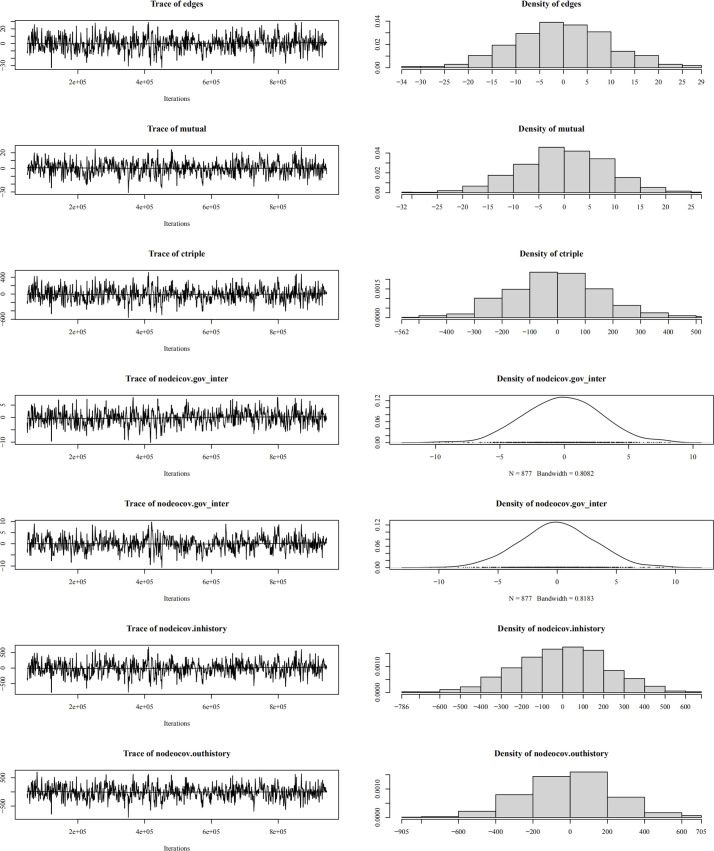
Convergence diagnostics for provincial-provincial ERGM.

**Fig 8:**
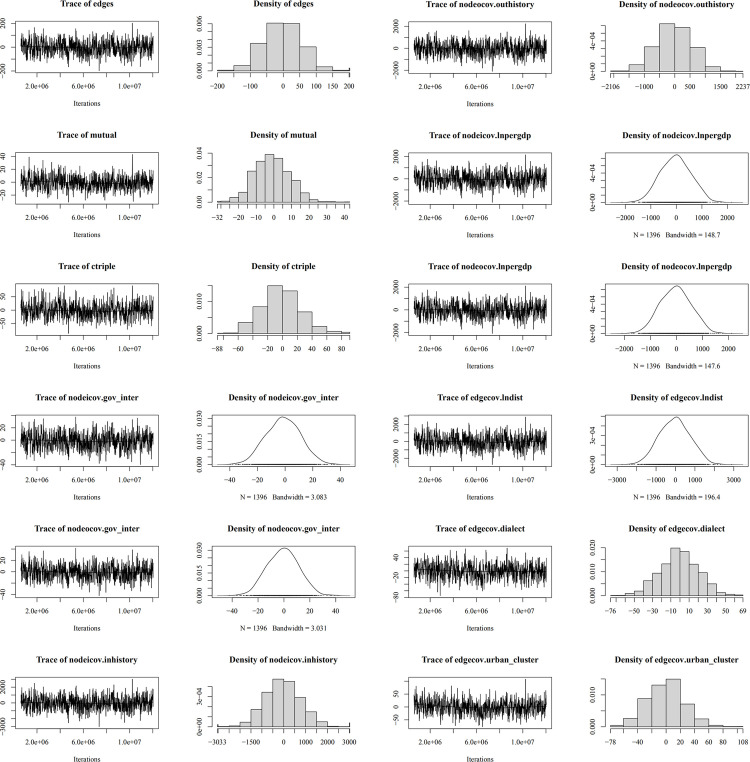
Convergence diagnostics for city-city ERGM.

**Fig 9:**
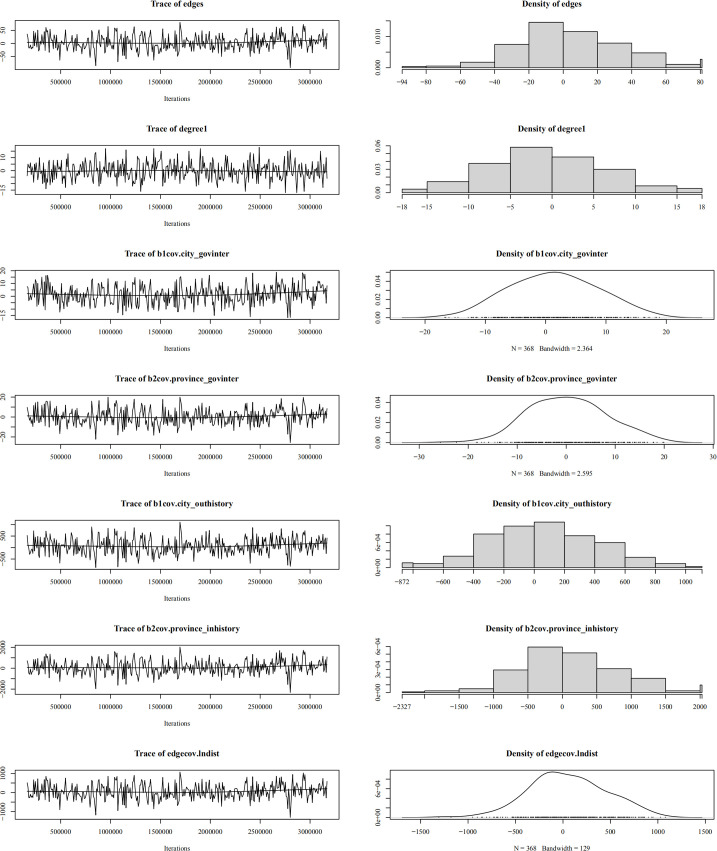
Convergence diagnostics for provincial-city ERGM.

**Fig 10:**
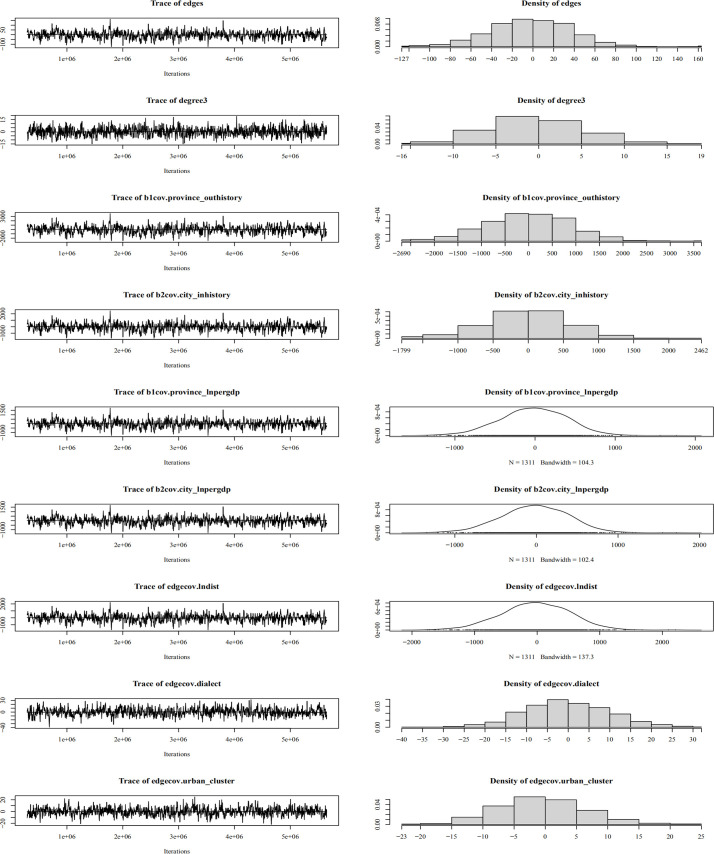
Convergence diagnostics for city-provincial ERGM.

Regarding convergence diagnostics (Fig 7–10), trace plots for the optimal model reveal the MCMC convergence of each variable. Effective simulations should avoid prolonged flat segments or sustained unidirectional trends, with all variables converging toward the horizontal reference line. The actual time-series plots resemble “white noise”, indicating well-calibrated parameters and satisfactory model convergence.

#### 4.2.3. Regional heterogeneity analysis.

We categorized the study regions into eastern, central, western, and northeastern China according to the national 14th Five-Year Plan, with these four macro-regions demonstrating significant political, economic and cultural disparities. Then we conducted separate analyses on the determinants of various types of HCC networks in China’s eastern, central, western, and northeastern regions. With the exception of provincial-city HCC models, which exhibited model degeneracy, other three HCC networks demonstrated significant regional heterogeneity in certain indicators.

For provincial-provincial HCC networks (Table 8), the eastern region demonstrates particularly strong significance for both *History of HCC establishment* of operation and origin place compared to other regions, highlighting how historical factors exert more pronounced influence on provincial-provincial HCC formation in eastern China.

**Table 8:** Regional heterogeneity analysis results (provincial-provincial HCCs).

Category	Variables	Eastern China	Central China	Western China	Northeast China
Endogenous structure	*Edges*	2.781*** (0.698)	3.213*** (1.113)	1.718** (0.708)	0.929 (2.022)
	*Mutual*	0.251 (0.223)	0.253 (0.223)	0.264 (0.225)	0.239 (0.214)
	*Ctriple*	0.045*** (0.011)	0.047*** (0.011)	0.062*** (0.012)	0.046*** (0.011)
Node covariates of operation place	*Government intervention*	−0.584 (1.178)	1.267 (1.535)	−0.054 (0.653)	1.366 (2.503)
	*History of HCC establishment*	0.042** (0.018)	0.001 (0.041)	0.010 (0.021)	0.093 (0.068)
Node covariates of origin place	*Government intervention*	−3.698*** (1.152)	−5.617*** (1.514)	−1.188 (0.752)	−4.900* (2.535)
	*History of HCC establishment*	−0.039** (0.016)	−0.090*** (0.021)	−0.090*** (0.016)	−0.066 (0.043)
AIC		1102	1093	1068	1106
BIC		1136	1126	1102	1140

Note: Dependent variable is provincial-provincial HCC connection, with 1 indicating a connection and 0 otherwise. BIC is larger than AIC, and the smaller the two values, the better the model fit. SEs appear in parentheses. Significance levels: * **p* *< 0.1, ** **p* *< 0.05, ***p < 0.01.

For city-city HCC networks (Table 9), Central China shows regional heterogeneity that is significantly different from other regions. First, the coefficient of *Government intervention* of origin place in the city-city HCC global regression was significantly negative, and it was only reflected in the central region in regional heterogeneity, indicating that the outward flow of HCCs in central cities was strongly influenced by government intervention factors. Second, the central region’s geographical centrality – being proximate to all other regions (unlike the eastern-west or western-northeast distances) – inverts the standard distance-decay pattern, resulting in a significantly positive *lndistance* coefficient for inter-city HCC establishment within this region.

**Table 9:** Regional heterogeneity analysis results (city-city HCCs).

Category	Variables	Eastern China	Central China	Western China	Northeast China
Endogenous structure	*Edges*	−3.935*** (0.033)	−3.857*** (0.033)	−3.782*** (0.034)	−3.776*** (0.032)
	*Mutual*	1.166*** (0.122)	1.213*** (0.121)	1.173*** (0.119)	1.205*** (0.120)
	*Ctriple*	0.562*** (0.061)	0.574*** (0.064)	0.582*** (0.063)	0.571*** (0.067)
Node covariates of operation place	*Government intervention*	0.659** (0.346)	0.295 (0.325)	0.450** (0.208)	1.247* (0.693)
	*History of HCC establishment*	−0.049*** (0.005)	−0.034*** (0.005)	−0.011** (0.005)	−0.037*** (0.009)
	*lnpergdp*	0.584*** (0.051)	0.279*** (0.058)	0.234*** (0.047)	0.413*** (0.127)
Node covariates of origin place	*Government intervention*	0.307 (0.310)	−1.676*** (0.378)	0.018 (0.198)	−0.081 (0.759)
	*History of HCC establishment*	−0.053*** (0.006)	−0.003 (0.007)	−0.046*** (0.006)	−0.059*** (0.015)
	*lnpergdp*	−0.298*** (0.048)	−0.426*** (0.059)	−0.093* (0.048)	−0.019 (0.127)
Edge covariates	*lndistance*	−0.137*** (0.027)	0.162*** (0.031)	−0.086*** (0.031)	−0.280*** (0.062)
	*Dialect*	0.376*** (0.068)	0.302*** (0.075)	0.264*** (0.074)	0.450*** (0.143)
	*Urban cluster*	0.247*** (0.060)	0.427*** (0.050)	0.391*** (0.058)	0.334** (0.137)
AIC		18730	18969	18942	19003
BIC		18842	19080	19054	19114

Note: Dependent variable is city-city HCC connection, with 1 indicating a connection and 0 otherwise. BIC is larger than AIC, and the smaller the two values, the better the model fit. SEs appear in parentheses. Significance levels: * **p* *< 0.1, ** **p* *< 0.05, ***p < 0.01.

Regarding provincial-city HCC networks (Table 10), we observe anomalous coefficient of *Government intervention* of operation place magnitudes with inflated Standard Errors, suggesting potential model degeneracy. Therefore, we do not explain the results of this type of regional heterogeneity.

**Table 10:** Regional heterogeneity analysis results (provincial-city HCCs).

Category	Variables	Eastern China	Central China	Western China	Northeast China
Endogenous structure	*Edges*	−0.566 (0.379)	−0.019 (0.655)	1.422** (0.650)	−1.993*** (0.053)
	*Degree (1)*	3.053*** (0.293)	3.369*** (0.320)	3.977*** (0.357)	1.638*** (0.203)
Node covariates of operation place	*Government intervention*	10.443*** (1.022)	22.077*** (6.289)	−1.322*** (0.483)	13.835* (7.551)
	*History of HCC establishment*	−0.074*** (0.008)	−0.316*** (0.065)	−0.057*** (0.007)	−0.199** (0.100)
Node covariates of origin place	*Government intervention*	−2.154** (0.848)	−2.302** (0.981)	0.995*** (0.307)	3.152*** (0.656)
	*History of HCC establishment*	−0.053*** (0.010)	−0.071*** (0.015)	−0.102*** (0.012)	−0.058*** (0.021)
Edge covariates	*lndistance*	−0.136*** (0.027)	−0.095** (0.047)	−0.182*** (0.044)	0.022*** (0.005)
AIC		6151	6329	6271	4930
BIC		6199	6377	6319	4979

Note: Dependent variable is provincial-city HCC connection, with 1 indicating a connection and 0 otherwise. BIC is larger than AIC, and the smaller the two values, the better the model fit. SEs appear in parentheses. Significance levels: * **p* *< 0.1, ** **p* *< 0.05, ***p < 0.01.

For city-provincial HCC networks (Table 11), the most pronounced regional heterogeneity manifests in the *lnpergdp* effects. Due to the relatively small economic gap within the eastern region, HCC establishment in the east is not significantly affected by the economic level.

**Table 11:** Regional heterogeneity analysis results (city-provincial HCCs).

Category	Variables	Eastern China	Central China	Western China	Northeast China
Endogenous structure	*Edges*	2.582*** (0.837)	2.589** (1.263)	0.763 (0.865)	0.665 (0.903)
	*Degree (3)*	5.485*** (0.409)	4.870*** (0.437)	4.269*** (0.337)	4.318*** (0.352)
Node covariates of operation place	*Government intervention*	−0.001 (0.008)	−0.018** (0.008)	−0.008 (0.007)	−0.007 (0.007)
	*History of HCC establishment*	−0.011 (0.066)	−0.037 (0.098)	0.021*** (0.064)	0.219*** (0.068)
Node covariates of origin place	*Government intervention*	−0.034*** (0.006)	0.188*** (0.012)	0.187*** (0.010)	0.187*** (0.010)
	*History of HCC establishment*	0.044 (0.060)	−0.577*** (0.104)	−0.221*** (0.064)	−0.211*** (0.068)
Edge covariates	*lndistance*	−0.209*** (0.039)	−0.183*** (0.044)	−0.236*** (0.041)	−0.237*** (0.040)
	*Dialect*	−0.257** (0.127)	−0.076 (0.133)	−0.567*** (0.152)	−0.568*** (0.146)
	*Urban cluster*	−0.579*** (0.178)	−1.287*** (0.283)	−0.441** (0.202)	−0.446** (0.196)
AIC		10003	9558	8950	8952
BIC		10066	9621	9013	9015

Note: Dependent variable is city-provincial HCC connection, with 1 indicating a connection and 0 otherwise. BIC is larger than AIC, and the smaller the two values, the better the model fit. SEs appear in parentheses. Significance levels: * **p* *< 0.1, ** **p* *< 0.05, ***p < 0.01.

## 5. Conclusions and policy implications

Under the context of enhancing China’s domestic economic circulation, China’s HCCs have played a unique role in facilitating regional economic cooperation compared to other social organizations. However, existing research lacks spatial network perspectives, and the underlying heterogeneous determinants remain systematically unexplored. Using establishment data of provincial-provincial, city-city, provincial-city, and city-provincial HCCs by the end of 2022, we apply SNA with GIS to characterize their distinct network spatial patterns. ERGM is then employed to analyze HCC connection determinants. Key findings include:

(1) This study is the first to reveal the spatial topological characteristics of China’s HCC networks. Point degree centrality in eastern China is high, but spatial patterns vary across network types. According to high node in-degree and out-degree centrality combined with its geographical spatial distribution, specific performance is as follows: provincial-provincial and provincial-city types exhibit a “rhombus-net” pattern, the city-city type follows a “small-ring-line” structure, and the city-provincial type forms a “large-ring-net” type. Guangdong, Fujian, Zhejiang, Jiangxi, and Shandong have always occupied core areas, which constitute the primary core hubs of China’s HCC networks. Provincial-provincial and city-city HCCs mainly flow within core areas and core areas flows to periphery areas, while provincial-city and city-provincial HCCs flow mutually between core and periphery areas.
(2) First, stronger government intervention and earlier HCC connections significantly suppress both provincial-provincial and city-city HCC establishment. This may relate to administrative barriers and traditional industry path dependence. However, this suppression show no statistical significance for provincial-city and city-provincial HCCs. We speculate that this is because central policies (e.g., Western Development Initiative, Xinjiang Aid Policy) that transcend local government, guiding interregional HCC flows. Importantly, this finding contrasts with prior studies emphasizing government support roles, such as officials facilitating HCCs through letters or cross-regional engagements [16–18]; Second, only city-city HCCs exhibit economic sensitivity, with less-developed origin cities favoring stronger commercial destinations. Provincial-level HCCs remain economically neutral; Third, road, dialect, and urban cluster distances significantly affect HCC establishment at the city-level place of operation but show no significant impact on the provincial-level place of operation.

Based on the above conclusions, this paper puts forward three policy recommendations:

(1) Although Beijing, Shanghai, Guangdong and other places are outstanding in the absorption of HCCs, and Zhejiang and Fujian are outstanding in the radiation of HCCs, the centrality and matching of these high nodes are not strong. According to the spatial organization theory of node source and sink in the flow space economic network [6], when the centrality of a node’s access degree is high, the node’s influence is strong. Therefore, local government should actively promote two-way geographical process of HCC flows in of the core node, play a reciprocal effect, and strengthen the influence of the core node.
(2) Optimize methods of government intervention. At the provincial-provincial and city-city levels of HCCs, local governments should reduce direct interference, avoiding excessive approval processes or local protectionism that may hinder the organic HCC establishment. Encourage the development of regional urban agglomeration, leveraging the role in weakening local protectionism, and guide HCCs to form multi-center agglomerations across broader national ranges. Utilize national regional macro-policies to facilitate more cross-level HCCs (such as provincial-city and city-provincial) that can compensate for market failures and effectively guide the rational flow of resource allocation.
(3) Dynamically adjust HCC development strategies. For early-established HCCs, governments should regularly assess their economic contributions and promote industrial upgrading (e.g., transitioning from trade-oriented to R&D-oriented models) to prevent over-reliance on traditional patterns that could weaken the flow of emerging industry-focused HCCs in the future. Since road distance affects the establishment of city-level HCCs, governments should optimize intercity transportation networks to reduce cross-regional collaboration costs for businesses.

## 6. Limitations and future research

This study provides a new perspective for understanding inter-regional economic and social linkages in China by analyzing the spatial network patterns and influencing factors of HCCs. Nevertheless, several limitations remain that offer avenues for future research.

(1) The spatial units of analysis in this study are limited to prefecture-level cities and higher administrative divisions in China. Due to constraints of article length and data availability, regional- and county-level HCCs fall outside the scope of this research.
(2) The use of ERGM allows the analysis of relational patterns and statistically significant associations, but it is inherently limited to static cross-sectional data and cannot capture how HCC networks evolve over time. Future studies could apply dynamic network models, such as Stochastic Actor-Oriented Model (SAOM) or TERGM, to explore how HCC formation responds to institutional reforms, market changes, or migration patterns.
(3) While the variable selection in this study is grounded in existing theoretical and empirical literature, the results are correlational in nature. Establishing causal relationships between HCC formation and its antecedents will require quasi-experimental designs, such as difference-in-differences or instrumental variable approaches.
(4) Although this study defines spatial clusters based on administrative flows, further research could apply community detection algorithms—such as modularity optimization or the Louvain method—to reveal potential emergent subgroups and assess their alignment with administratively defined clusters. In addition, statistical validation techniques could be explored to enhance the robustness of bipartite network structures [38,39].
